# Advanced Stage IVb Classical Hodgkin’s Lymphoma in a 9/11 First Responder: The Persistent Health Risks and Impact of Environmental Exposures

**DOI:** 10.7759/cureus.93417

**Published:** 2025-09-28

**Authors:** Tanmai Bandarupalli, Syed A Hasan, Mariah George, Yanisa Del Toro

**Affiliations:** 1 College of Medicine, University of Central Florida College of Medicine, Orlando, USA; 2 Internal Medicine, University of Central Florida College of Medicine, Orlando, USA

**Keywords:** 9/11, classic hodgkin lymphoma, occupational exposure, skeletal metastases, world trade center

## Abstract

This case report details the clinical journey of a first responder from the events of 9/11 who presented with significant B symptoms and right hip pain, ultimately diagnosed with stage IVb classical Hodgkin’s lymphoma with extensive metastasis. Imaging revealed a right proximal femoral mass, mediastinal and supraclavicular nodal metastases, and multiple hypermetabolic splenic masses, emphasizing the aggressive nature of the disease and the importance of early detection and prompt management. Following referral to an oncologist, the patient was treated with six cycles of the chemotherapy regimen consisting of brentuximab plus doxorubicin, vinblastine, and decarbazine (brentuximab-AVD) without the need for surgical excision. A follow-up PET/CT scan seven months after diagnosis showed no active tumors, indicating that the patient was in remission. Three years following the initial diagnosis, the patient displays no signs of recurrence and continues to remain B-symptom-free. He does report lingering intermittent and stable right hip pain managed with medications as needed.

## Introduction

More than 20 years after the devastating events of September 11, 2001, the health consequences for those exposed to World Trade Center (WTC) dust continue to manifest, particularly among the first responders. Thousands of these individuals, along with many bystanders, suffer from a range of health issues, including acute traumatic injuries, respiratory and digestive disorders, mental health conditions, and an increased risk of cancer [1].

As more time passes, cancer development persists as a serious concern for first responders as reports now show that New York City Fire Department (FDNY) cancer deaths surpass casualties of the WTC attacks [2]. Studies have shown that 9/11 responders face a 25% increased risk of prostate cancer, a 219% increased risk of thyroid cancer, and a 41% increase in leukemia [2]. This is in part due to the presence of carcinogens such as asbestos, polychlorinated biphenyls, benzenes, and dioxins identified in the WTC dust [2]. However, increased survival rates among this cohort have been attributed to enhanced detection methods and access to comprehensive healthcare programs, such as the World Trade Center Health Program and the September 11th Victim Compensation Fund, which have been extended until 2090 by the Zadroga Act of 2015 [1].

Classical Hodgkin's lymphoma remains the leading lymphoma cancer diagnosis in young adults. The Epstein-Barr virus, widely known as a causative agent of infectious mononucleosis, is highly associated with the development of classical Hodgkin's lymphoma. Genetics and immunodeficiencies also play a role in susceptibility and have been well-researched in the literature. However, currently, there is little evidence to support the role of environmental risk factors in the development of this lymphoma [3].

## Case presentation

Initial presentation and workup

This case centers around a male patient in his 50s with a history of hypertension who presented to the clinic with complaints of nighttime fevers (99-101°F), night sweats, and significant unintentional weight loss of approximately 20 pounds over 1.5 months. His clinical and diagnostic course spans over the next three months (Table 1). He initially also reported new-onset significant right hip pain, which was constant but alleviated by acetaminophen (Tylenol) and ibuprofen. The patient denied any recent trauma or falls. Upon physical examination, right lower quadrant abdominal pain was noted along with point tenderness on the lateral aspect of the femur. Lymphadenopathy was not appreciable due to large body habitus. Recent past medical history includes a throat infection treated with montelukast and a tooth infection treated with amoxicillin. He was also noted to be deficient in vitamin D and iron, for which he was taking supplements. His medical history was notable for a normal colonoscopy and endoscopy the previous year, although he had been treated for *Helicobacter pylori* infection at that time. Initial laboratory investigations included low-density lipoprotein (LDL), C-reactive protein (CRP), complete blood count (CBC), comprehensive metabolic panel (CMP), and orders for hip and femur imaging. Low-density lipoprotein (LDL) cholesterol was found to be normal at 158 mg/dL, while complete blood count (CBC) was within normal limits, including values of a white blood cell (WBC) count of 8.2 cells/mm³, hemoglobin of 12.7 g/dL, and platelets at 267,000/μL. Comprehensive metabolic panel (CMP), including liver function tests (LFTs), was also reported to be normal.

**Table 1:** Timeline of Clinical Evaluation and Management HTN: Hypertension; LDL: low-density lipoprotein; CRP: C-reactive protein; CBC: complete blood count; CMP: comprehensive metabolic panel; SUV: standardized uptake value

Date	Event	Findings
Initial Visit	Clinic presentation	Male in 50s with HTN presenting with night fevers (99-101°F), night sweats, 20 lb weight loss over 1.5 months, right hip pain. Physical exam: right lower quadrant abdominal pain, femur tenderness. Labs: LDL, CRP, CBC, CMP ordered.
Month 1 (Day 1)	Imaging: X-ray right hip and femur	X-rays unremarkable, no defects detected.
Month 1 (Day 11)	Imaging: MRI right hip	MRI showed infiltrative intramedullary mass lesion in right femoral neck with extensive edema, suggestive of lymphoma, multiple myeloma, or metastasis.
Month 2	Imaging: CT chest/abdomen/pelvis	CT chest: extensive mediastinal and supraclavicular nodal metastases. CT abdomen/pelvis: multiple splenic masses, retroperitoneal lymphadenopathy, periportal mass with duodenal mass effect. Osseous lesion in right proximal femur consistent with metastasis.
Month 3	Imaging: PET/CT skull to thigh	Multiple hypermetabolic lymphadenopathy and masses: supraclavicular, subclavicular, mediastinal, splenic, abdominal lymph nodes, and right femur lesion. SUVs ranging from 1.2 to 35.4.
Month 3	Biopsy and pathology	Biopsy: atypical lymphoid infiltrate, classical Hodgkin/Reed-Sternberg cells. IHC positive for CD15, CD30; weak CD20, PAX5, PD-L1. Diagnosis: Stage IVb classical Hodgkin’s lymphoma.
Months 3-9	Treatment: chemotherapy (Brentuximab-AVD)	Port placement for chemotherapy. Six planned cycles of brentuximab plus doxorubicin, vinblastine, dacarbazine.
Months 5 and 6	Follow-up imaging	Restaging PET/CT after three chemo cycles: no hypermetabolism detected.
Month 9	End of chemotherapy	Final PET/CT three weeks after last cycle showed resolution of metabolically active areas.
3 years post diagnosis	Long-term follow-up	No recurrence or B symptoms. Fully returned to baseline physical activity. Reports intermittent stable right hip pain controlled with medication.

Month 1: initial imaging

**Figure 1:**
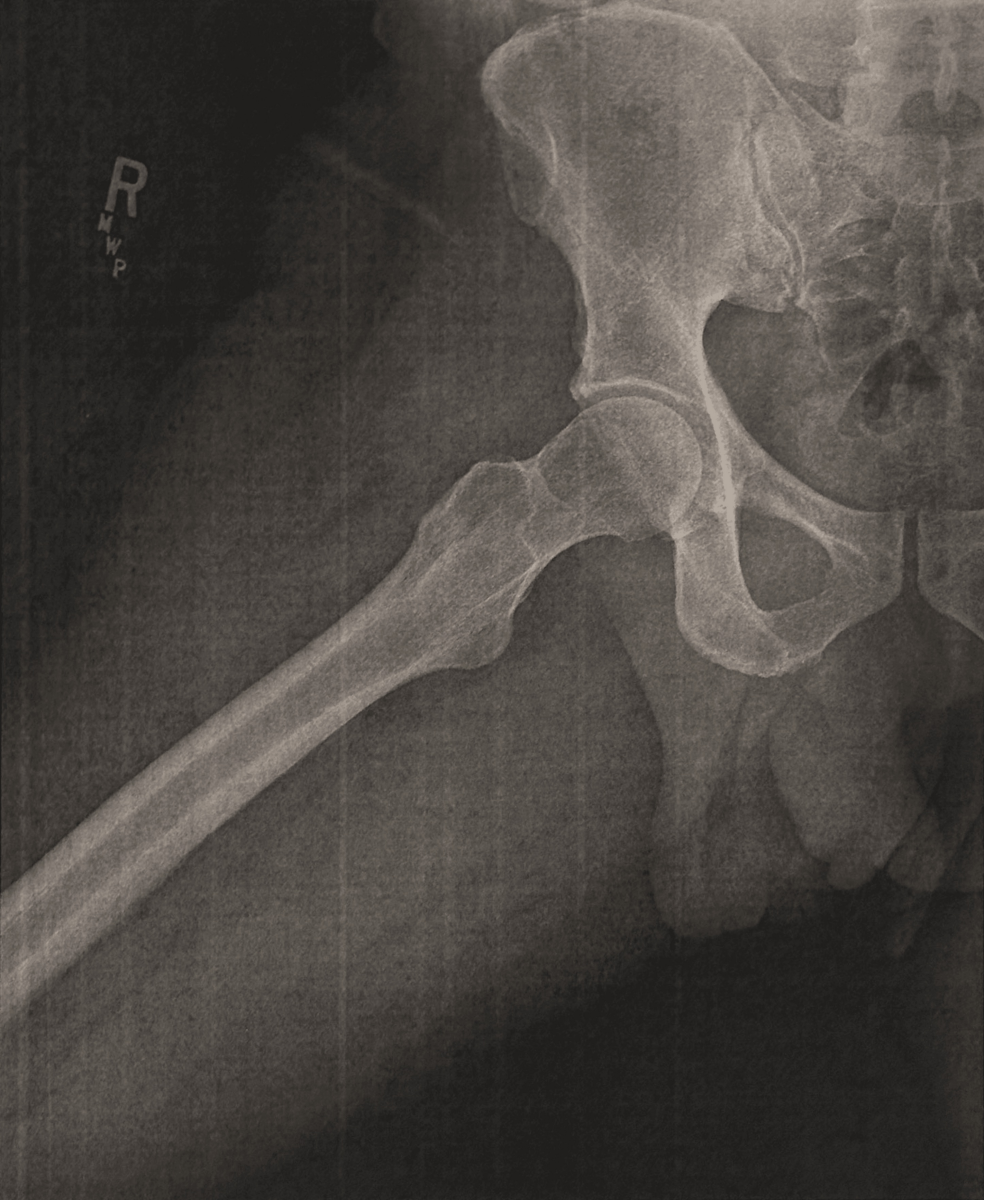
X-Ray of the Right Hip (Month 1) No significant clinical findings.

**Figure 2:**
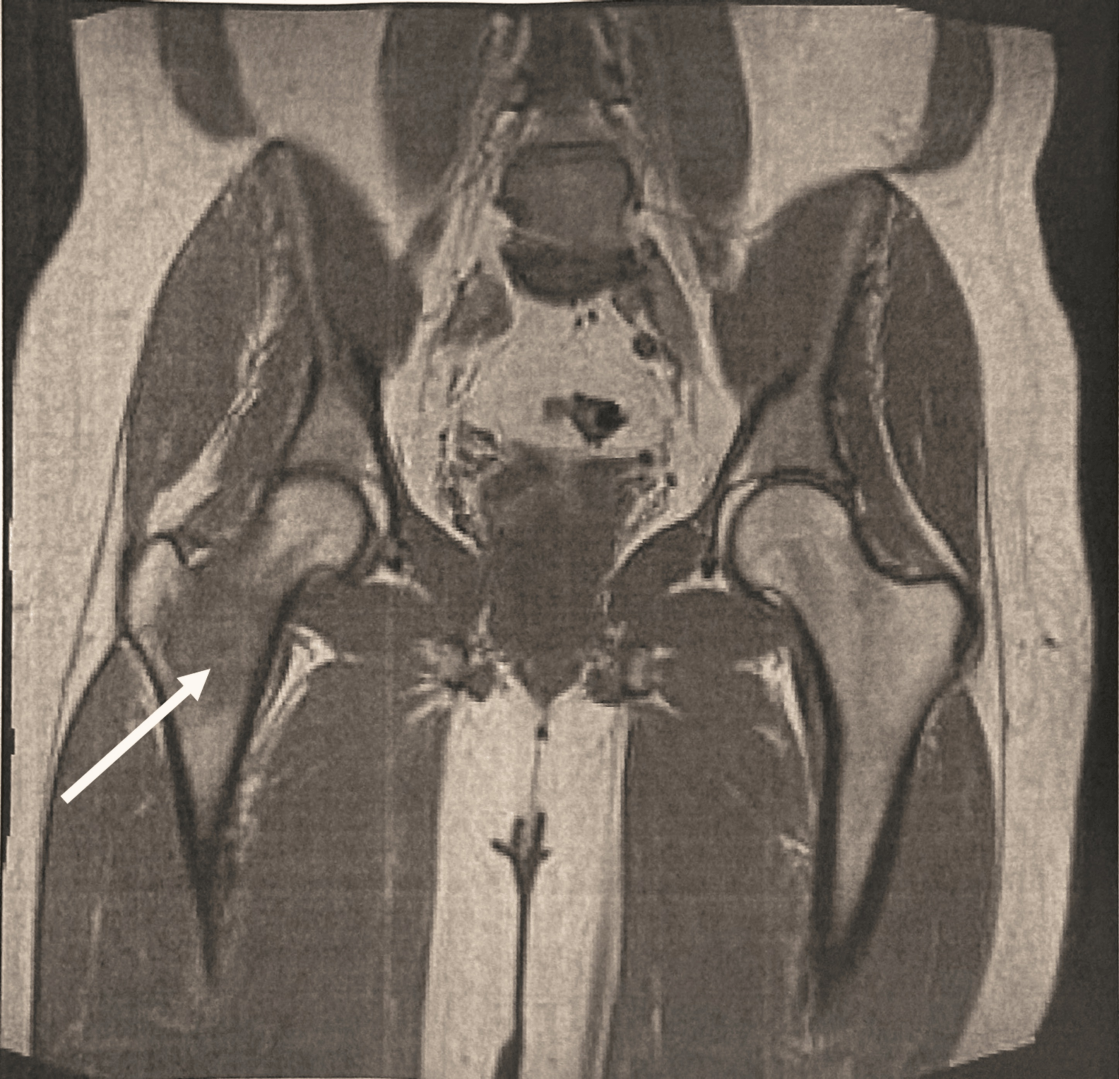
MRI of the Right Hip (Month 1) Infiltrative, intramedullary-based mass lesion within the right femoral neck.

The following day, the patient presented to obtain X-rays of the right hip and femur. Both imaging results were unexpectedly unremarkable, showing no defects and resulting in a normal impression (Figure 1). Despite this initial evaluation, the patient continued to have worsening symptoms that eventually culminated in further imaging consisting of a right hip magnetic resonance imaging (MRI) without contrast 11 days later. The MRI illustrated concerning findings of an infiltrative, intramedullary-based mass lesion within the right femoral neck (Figure 2). Accompanying extensive intraosseous and perilesional edema was also present. This presentation is typically oncological in nature, with the leading diagnosis at the time revolving around lymphoma, multiple myeloma, or another metastatic process. This finding warranted further imaging.

Month 2: advanced imaging and initial diagnosis

**Figure 3:**
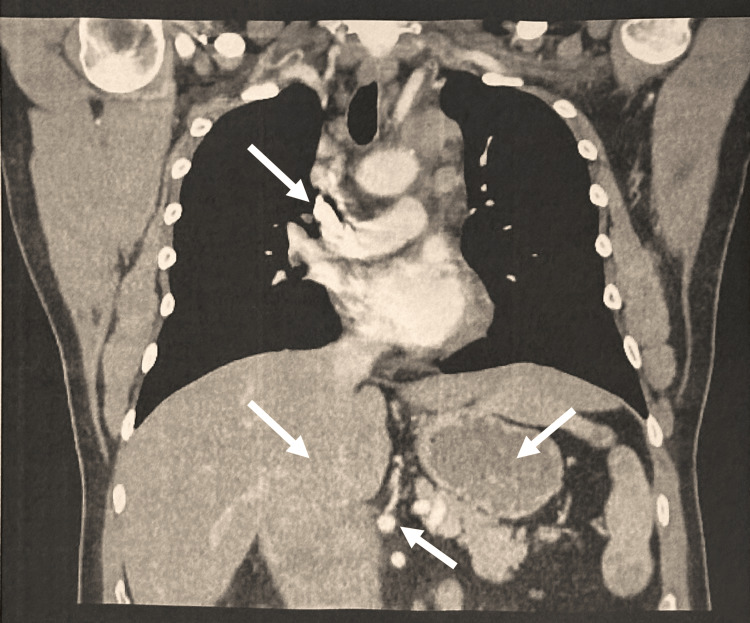
CT Chest (Month 2) Extensive mediastinal and supraclavicular nodal metastases.

To assess for a metastatic process, a computed tomography (CT) scan of the chest, abdomen, and pelvis was performed. The CT scan of the chest revealed extensive mediastinal and supraclavicular nodal metastases (Figure 3). Additionally, the CT abdomen and pelvis showed multiple splenic masses, enlarged retroperitoneal lymph nodes, and a well-circumscribed periportal mass causing a mass effect on the second part of the duodenum and adjacent vessels, which likely represented enlarged lymph nodes. However, a duodenal malignancy could not be ruled out. An osseous lesion was identified in the right proximal femur, consistent with metastasis. A bone scan was recommended, and a biopsy was suggested for definitive diagnosis.

Month 3: further workup and diagnosis

**Figure 4:**
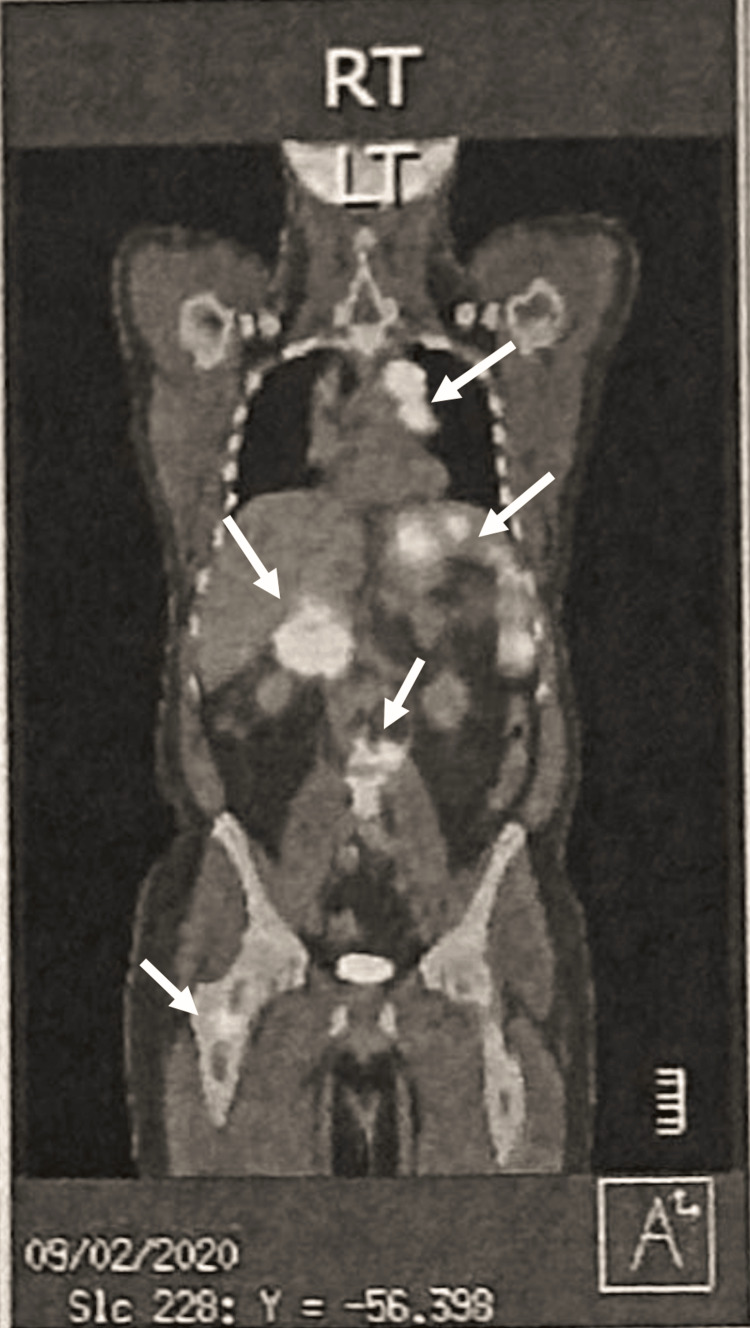
PET-CT Skull to Thigh (Month 3) Right supraclavicular lymph node with an SUV of 4.1; right subclavicular lymphadenopathy with an SUV of 3.2; substantial perivascular mediastinal mass with SUV of 35.4; mediastinal lymphadenopathy with with an SUV of 1.2; hypermetabolic right paratracheal lymph node with an SUV of 4.4; multifocal hypermetabolic splenic masses with an SUV of 19.8; hypermetabolic upper abdominal lymphadenopathy in the portacaval and mesenteric root distribution with an SUV of 28.1; right intertrochanteric femur lesion with an SUV of 7.5.

In month 3, following the patient’s initial clinic visit, a positron emission tomography/computed tomography (PET/CT) scan provided more detailed imaging that revealed significant findings (Figure 4). In the head and neck region, a 1.2 cm right supraclavicular lymph node with a standardized uptake value (SUV) of 4.1 was identified. The thoracic region showed a 1x0.8 cm right subclavicular lymphadenopathy with an SUV of 3.2, along with a substantial perivascular mediastinal mass measuring 5.5x3.2 cm, which exhibited a high SUV of 35.4. Additional findings included mediastinal lymphadenopathy with a 2.2x1.3 cm subcarinal/right parasagittal lymphadenopathy with an SUV of 1.2 and a mildly hypermetabolic right paratracheal lymph node with an SUV of 4.4. The abdomen and pelvis displayed multifocal hypermetabolic splenic masses, the largest of which measured 5x5 cm with an SUV of 19.8. Furthermore, hypermetabolic upper abdominal lymphadenopathy was noted in the portacaval and mesenteric root distribution, with the largest nodal mass in the portacaval region measuring 7x6.2 cm with an SUV of 28.1. In the osseous structures, a hypermetabolic lesion was identified in the right intertrochanteric femur, extending to the base of the femoral neck, with an SUV of 7.5.

These findings were highly suggestive of lymphomatous involvement, prompting a CT-guided core excisional biopsy of the retroperitoneal lymph node. Biopsy findings included atypical lymphoid infiltrate in a dense fibrotic background composed of scattered large cells, resembling the classical Hodgkin's/Reed-Sternberg cells. Immunohistochemistry of the biopsy contributed to the diagnosis of Stage IVb classical Hodgkin’s lymphoma, being positive for markers CD15 and CD30, and weakly positive for CD20, paired box protein 5 (PAX5), and programmed death-ligand 1 (PD-L1). Concurrent flow cytometry revealed no monoclonal B cells or abnormal T cells, further supporting the diagnosis.

Treatment

The patient was promptly referred to an oncologist and orthopedic surgeon who initiated a treatment plan that consisted of six cycles with the chemotherapy cocktail brentuximab plus doxorubicin, vinblastine, and dacarbazine (brentuximab-AVD) without any necessity for surgical excision. A chemotherapy port was placed, and the patient began receiving regular treatments. The patient's treatment course was well-tolerated, and within two months of initiating treatment, he reported significant improvement in his symptoms, including the improvement of his hip pain and the resolution of his B symptoms of night sweats and fever. He reported initial fatigue and diarrhea, followed by hair loss, all of which are common side effects of chemotherapy.

Outcome and follow-up

Restaging PET/CT scan completed after three cycles of chemotherapy showed no hypermetabolism, indicating that the patient was in remission. The patient continued with the chemotherapy regimen, with the sixth and final cycle completed nine months following initial diagnosis. A PET/CT performed three weeks after the final cycle of chemotherapy showed resolution of previously identified metabolically active areas. Follow-up CTs were completed four months, seven months, one year, and two years after completion of chemotherapy, which were all negative for malignancy recurrence. Three years following initial diagnosis, the patient displays no signs of recurrence and continues to remain B-symptom-free. He has fully returned to his baseline physical activity tolerance but does describe mild peripheral numbness that does not limit his activities of daily living (ADLs). He reports lingering intermittent and stable right hip pain, mitigated with medications as needed.

## Discussion

This case illustrates the intricate relationship between environmental exposure, such as that experienced by 9/11 first responders, and the development of aggressive malignancies like classical Hodgkin’s lymphoma (cHL). The patient’s presentation with B symptoms and right hip pain comes as an example of how cHL can manifest, but it also emphasizes how crucial it is to consider a patient’s occupational history when assessing potential malignancies. Interestingly, currently, little evidence exists in the literature for environmental factors leading to the development of classical Hodgkin’s lymphoma [3].

The extensive disease revealed through imaging, including widespread lymphadenopathy and splenic involvement, reflects the aggressive nature of cHL when diagnosed at an advanced stage. The prompt identification and confirmation of the diagnosis through biopsy and immunohistochemistry allowed for a positive response and eventual remission. Typically, with classical Hodgkin's lymphoma, there is an estimated cure rate of 90%, with an estimated 10%-25% having relapse or refractory disease despite first-line treatment following a complete remission [3].

The patient’s successful outcome demonstrates that, even in advanced stages, cHL can be effectively treated with appropriate chemotherapeutic care. The intersection of the patient’s history as a 9/11 responder and the development of cHL serves as a reminder of the lasting health impacts of environmental disasters and the need for ongoing vigilance in monitoring those at risk. Earlier detection and free level of care to first responders via the Zadroga Act of 2015 has alleviated the mortality rates, which are much lower than the incidence rates [1,4]. Despite this, more than 23 years after the tragic events of 9/11, more firefighters have now died due to illnesses developed after the incident [5,6].

When performing retrospective case studies, especially on unique situations, there tend to be limitations due to their inherent nature, including limited generalizability and confounding factors. One major limitation specific to this case report is recall bias, particularly the potential overemphasis on the rare event of 9/11. The Bradford Hill criteria can be used to evaluate the association between exposure and progression of Hodgkin's lymphoma in this patient. For example, temporality is established, as the exposure occurred years prior to cancer diagnosis. The presence of known carcinogens in the dust, including benzene and dioxins [2], supports biological plausibility. While direct evidence linking WTC exposure to Hodgkin lymphoma is limited, studies have reported increased rates of malignancies among exposed populations. The patient himself reports that nearly every member of his squad of first responders has received a cancer diagnosis over the past 23 years since the events of 9/11. In this patient, it is unknown the extent to which genetic susceptibility or other past medical history, such as possible past exposure to EBV, contributed to the cancer diagnosis [3]; therefore, the strength and consistency of this association remain uncertain.

## Conclusions

With over 400,000 bystanders exposed to toxic dust from the WTC attacks and more than 34,000 confirmed cases of various health conditions, including cancer, among survivors, this case report highlights the importance of screening for this uniquely vulnerable population. Outlined in this report was a first responder’s sudden and advanced initial diagnosis of stage IVb classical Hodgkin’s lymphoma with extensive metastasis. Despite the severity of the condition, prompt and appropriate management led to complete remission and resolution of symptoms. While we cannot confirm that WTC dust exposure was the direct cause of this patient's lymphoma, this case highlights the need for careful screening and high clinical suspicion in this population. When all factors impacting a patient’s health are considered, the potential for favorable outcomes is increased and can accelerate the identification and treatment of similar cases.

## References

[1] (2025). World Trade Center Health Program. https://www.cdc.gov/wtc/.

[2] Shapiro MZ, Wallenstein SR, Dasaro CR (2020). Cancer in general responders participating in World Trade Center Health programs, 2003-2013. JNCI Cancer Spectr.

[3] Brice P, de Kerviler E, Friedberg J (2021). Classical Hodgkin lymphoma. Lancet.

[4] Kehm RD, Li J, Takemoto E, Yung J, Qiao B, Farfel MR, Cone JE (2023). Mortality after the 9/11 terrorist attacks among world trade center health registry enrollees with cancer. Cancer Med.

[5] Zeig-Owens R, Webber MP, Hall CB (2011). Early assessment of cancer outcomes in New York City firefighters after the 9/11 attacks: an observational cohort study. Lancet.

[6] Katersky A Katersky A, More FDNY members have died from World Trade Center illnesses than killed on 9/11, ABC News, September 9, 2024. https://abcnews.go.com/US/fdny-members-died-world-trade-center-illnesses-killed/story?id=113517792.

